# Perioperative Goal-Directed Therapy Using Invasive Uncalibrated Pulse Contour Analysis

**DOI:** 10.3389/fmed.2018.00012

**Published:** 2018-01-30

**Authors:** Bernd Saugel, Daniel A. Reuter

**Affiliations:** ^1^Department of Anesthesiology, Center of Anesthesiology and Intensive Care Medicine, University Medical Center Hamburg-Eppendorf, Hamburg, Germany; ^2^Department of Anesthesiology and Intensive Care Medicine, University Medical Center Rostock, Rostock, Germany

**Keywords:** hemodynamic monitoring, cardiac output, stroke volume, pulse pressure variation, stroke volume variation, pulse wave analysis

## Abstract

“Perioperative goal-directed therapy” (PGDT) aims at an optimization of basic and advanced global hemodynamic variables to maintain adequate oxygen delivery to the end-organs. PGDT protocols help to titrate fluids, vasopressors, or inotropes to hemodynamic target values. There is considerable evidence that PGDT can improve patient outcome in high-risk patients if both fluids and inotropes are administered to target hemodynamic variables reflecting blood flow. Despite this evidence, PGDT strategies aiming at an optimization of blood flow seem to be not well implemented in routine clinical care. The analysis of the arterial blood pressure waveform using invasive uncalibrated pulse contour analysis can be used to assess hemodynamic variables used in PGDT protocols. Pulse contour analysis allows the assessment of stroke volume (SV)/cardiac output (CO) and pulse pressure variation (PPV)/stroke volume variation (SVV) and thus helps to titrate fluids and vasoactive agents based on principles of “functional hemodynamic monitoring.” Pulse contour analysis-based PGDT treatment algorithms can be classified according to the hemodynamic variables they use as targets: PPV/SVV, SV/CO, or a combination of these variables. From a physiologic point of view, algorithms using both dynamic cardiac preload and blood flow variables as hemodynamic targets might be most effective in improving patient outcome. Future research should focus on the improvement of hemodynamic treatment algorithms and on the identification of patient subgroups in which PGDT based on uncalibrated pulse contour analysis can improve patient outcome.

## Background

“Perioperative goal-directed therapy” (PGDT), i.e., the assessment and goal-directed optimization of hemodynamic variables, might improve the quality of perioperative care and patient outcome. PGDT aims at an optimization of basic and advanced global hemodynamic variables to maintain adequate oxygen delivery to the end-organs. PGDT protocols help to titrate fluids, vasopressors, or inotropes to hemodynamic target values that can be assessed with different hemodynamic monitoring technologies.

The analysis of the arterial pressure waveform using uncalibrated pulse contour analysis can be used to estimate stroke volume (SV), cardiac output (CO), and dynamic variables of cardiac preload [pulse pressure variation (PPV), stroke volume variation (SVV)].

In this article, we will describe the physiological background and the clinical application of PGDT using invasive uncalibrated pulse contour analysis for the assessment of hemodynamic values.

## PGDT: A Gap Between Evidence and Clinical Practice

Numerous randomized-controlled trials and meta-analyses demonstrate that there is an increasing body of evidence that PGDT can contribute to an improvement in patient outcome (1–9) and guidelines and consensus statements recommend PGDT in major surgery patients (10–12).

A meta-analysis including 29 studies demonstrated that preemptive PGDT strategies targeting cardiac index (CI) or oxygen delivery improve patient outcome in terms of mortality and postoperative complications in moderate- and high-risk surgical patients (1).

In accordance, a meta-analysis including 32 randomized-controlled trials showed that protocol-based optimization of tissue perfusion in terms of optimization of hemodynamics decreases postoperative mortality and organ dysfunction in high-risk surgical patients, particularly when CI, oxygen delivery, and oxygen consumption are used to guide therapy (2).

Another meta-analysis confirmed that PGDT improves postoperative mortality and morbidity in high-risk surgical patients undergoing major non-cardiac surgery when fluids and inotropes are used to achieve CI or oxygen delivery target values (3).

A Cochrane meta-analysis including 31 randomized-controlled trials concluded that a perioperative increase in global blood flow to explicitly defined goals with fluids and/or inotropes reduces complications and length of hospital stay, but not mortality, in adult patients (4). An updated version of this Cochrane meta-analysis included in the paper reporting the OPTIMISE trial (13) provided further evidence that PGDT increasing global blood flow to explicitly defined goals reduces postoperative complications.

Despite the evidence that PGDT can improve postoperative outcome in high-risk patients undergoing major surgery, PGDT strategies aiming at an optimization of blood flow seem to be not well implemented in routine perioperative care. This is reflected by the fact that there is a wide variation in clinical practice and that in only about 10–20% of major non-cardiac surgery patients CO monitoring is used during perioperative care (14). Moreover, it has been shown that, in general, CO monitoring is used only by about one-third of anesthesiologists in Europe and the United States (15). Suggested explanations for the fact that advanced hemodynamic monitoring is rarely used in perioperative care include a lack of experience or knowledge regarding monitoring technologies and local factors such as a lack of available technical equipment or problems with reimbursement (14).

## Invasive Uncalibrated Pulse Contour Analysis: Basic Measurement Principles

**Figure 1:**
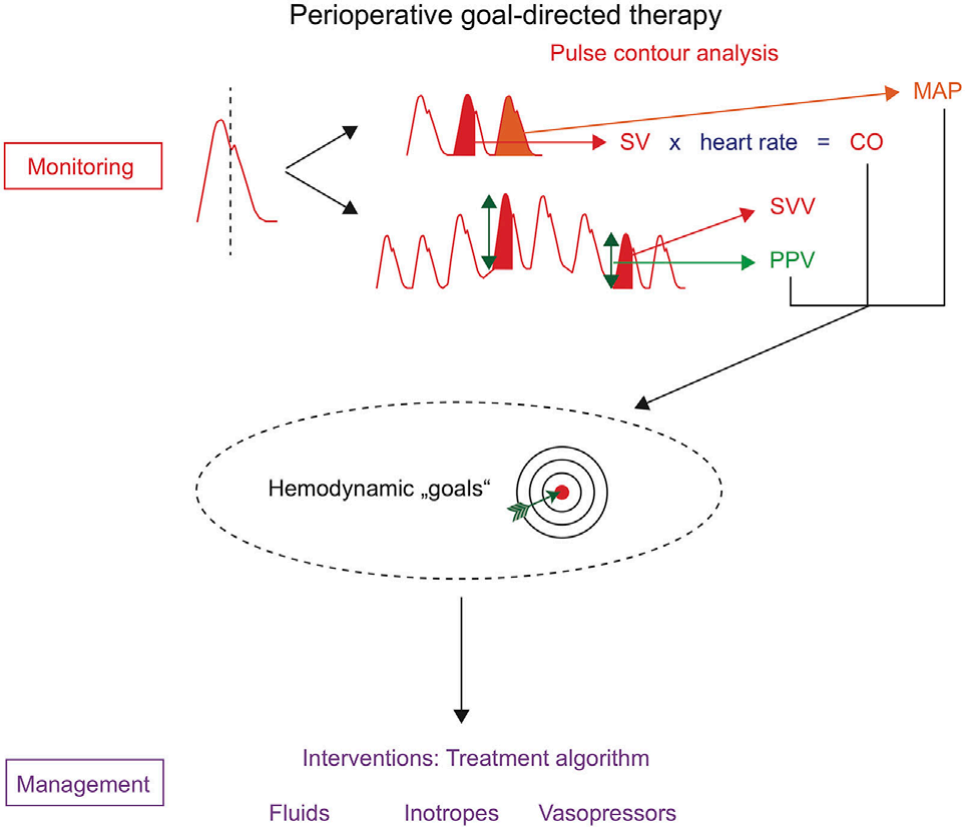
Perioperative goal-directed therapy based on uncalibrated pulse contour analysis. Hemodynamic monitoring with uncalibrated pulse contour analysis allows the assessment of mean arterial pressure (MAP), stroke volume (SV), cardiac output (CO), stroke volume variation (SVV), and pulse pressure variation (PPV). These hemodynamic variables can be used as hemodynamic “goals” in treatment algorithms that trigger therapeutic interventions (hemodynamic management).

One technique that can be used to assess hemodynamic variables for PGDT is invasive uncalibrated pulse contour analysis. The analysis of the arterial blood pressure waveform (pulse contour analysis) allows not only the monitoring of arterial blood pressure but also the estimation of SV, CO, and PPV/SVV (Figure 1). An arterial catheter is placed in most high-risk patients undergoing major surgery for invasive (“direct”) continuous arterial blood pressure monitoring and for point of care blood gas analysis. Therefore, pulse contour analysis can be used for PGDT without the need for the placement of additional intravascular catheters.

There are a variety of different algorithms for pulse contour analysis that enable SV to be estimated from the arterial blood pressure waveform (16, 17). These algorithms analyze the shape and characteristics of the waveform considering that the waveform is determined by left-ventricular SV and arterial impedance (i.e., ventriculo-arterial coupling). Other factors influencing pulse pressure and the arterial blood pressure waveform are the cardiac contractility, the vascular compliance, and the peripheral vascular resistance. Some hemodynamic monitors combine pulse contour analysis with a second CO measurement technique (e.g., transpulmonary thermodilution or lithium dilution) to calibrate the continuous pulse contour-derived CO signal to an independent external CO value (18). This external calibration increases the accuracy and precision of pulse contour-derived CO measurements, but also increases the invasiveness of the monitoring technology and is, therefore, recommended in patients with rapid changes in vasomotor tone that require frequent recalibration (19, 20). In the perioperative setting, however, uncalibrated pulse contour analysis only requiring an arterial catheter can be used. The term uncalibrated pulse contour analysis is misleading, because even uncalibrated systems perform an “autocalibration” of the CO signal (using data from large patient databases, biometric data, or characteristics of the arterial blood pressure waveform) (18).

Besides the estimation of SV, pulse contour analysis allows the assessment of dynamic cardiac preload variables (PPV, SVV) that—based on heart-lung interactions during mechanical ventilation—can be used to predict fluid responsiveness (21).

Although pulse contour analysis can be easily used in patients with an arterial catheter, the method has several limitations that are crucial to know to avoid erroneous measurements. First, pulse contour analysis depends on an optimal arterial pressure signal. Therefore, to assure impeccable arterial blood pressure waveform recording, one has to meticulously avoid clotting of the arterial catheter, over- or underdamping of the tubing system, or incorrect zeroing of the pressure transducer and monitoring system. In addition, the clinical usefulness of pulse contour analysis is limited in patients with high-grade cardiac arrhythmias and rapid changes or profound abnormalities in vasomotor tone (e.g., in septic patients or patients with cardiocirculatory alterations due to advanced liver disease) (22). The use of PPV and SVV is limited to patients with sinus rhythm, mechanical ventilation, and tidal volumes ≥8 mL/kg predicted body weight. Of note, the capabilities of PPV to predict fluid responsiveness are limited for PPV values between 9 and 13% (gray zone for the prediction of fluid responsiveness) (23).

## How to Use Invasive Uncalibrated Pulse Contour Analysis for PGDT: Physiologic Background

The cardiac function curve (i.e., Frank–Starling curve) describes the relation of ventricular preload or left-ventricular end-diastolic pressure and SV. A left ventricle functioning on the steep part of the cardiac function curve will increase SV after an increase in cardiac preload (e.g., due to fluid administration). This state of “preload reserve” is clinically referred to as “fluid responsiveness,” i.e., an increase in blood flow following fluid administration. Because ventricular function is a major determinant of the shape of the cardiac function curve, fluid administration must be performed cautiously to avoid fluid overload and circulatory failure, especially in patients with poor ventricular function in whom the heart is already working on the flat part of the curve.

Based on these basic physiologic principles, pulse contour analysis provides crucial hemodynamic variables reflecting fluid responsiveness (PPV, SSV) and blood flow (SV, CO) that can be used in PGDT protocols to titrate fluids and vasoactive agents based on principles of “functional hemodynamic monitoring” (24). Functional hemodynamic monitoring using pulse contour analysis can be used to predict fluid responsiveness using the dynamic cardiac preload variables PPV or SVV and to assess the dynamic response to fluid administration using real-time CO monitoring. The diagnostic passive leg raising test, that was proposed to assess fluid responsiveness in critically ill patients (25), cannot be routinely performed intraoperatively and is usually not part of PGDT protocols. In addition to fluid therapy, pulse contour analysis enables vasopressors and inotropes to be titrated according to arterial blood pressure and SV/CO, respectively.

## How to Use Invasive Uncalibrated Pulse Contour Analysis for PGDT: Clinical Application

Invasive uncalibrated pulse contour analysis is frequently used for the assessment of hemodynamic variables within PGDT protocols (8, 9, 13, 26, 27). Numerous different algorithms for pulse contour analysis-based PGDT have been proposed.

These treatment algorithms can be classified according to the hemodynamic variables they use as targets: some algorithms are solely based on either dynamic cardiac preload variables (PPV, SVV) or blood flow variables (SV, CO/CI); other algorithms combine these dynamic cardiac preload and blood flow variables (9).

The OPTIMISE trial is an example for a study using pulse contour analysis solely to optimize blood flow (13). In this largest available multicenter randomized-controlled trial, uncalibrated pulse contour analysis was used to maximize SV with repetitive colloidal fluid boluses (250 mL over 5 min) (13). Maximal SV was defined “as the absence of a sustained rise in SV of at least 10% sustained for 20 min or more in response to a fluid challenge” (13). After the first fluid bolus, patients in the treatment group also received inotropic support (dopexamine in a fixed dose) to achieve the maximal value of SV (13). In the OPTIMISE trial, PPV or SVV were not part of the treatment algorithm. In the study group, the composite endpoint of predefined moderate or severe postoperative complications and mortality at day 30 after surgery occurred less frequently in the intervention group (36.6%) compared with the control group (43.4%), but this finding did not reach statistical significance (13).

Compared with the approach of maximizing SV by using the full cardiac preload reserve, PGDT algorithms targeting both dynamic cardiac preload parameters and SV/CO may help to better tailor the hemodynamic management to the individual patient (28, 29).

In the ongoing follow-up study of the OPTIMISE trial (OPTIMISE II^1^), SVV is included in the hemodynamic management protocol in addition to the SV target (fluid challenge not recommended if SVV is <5%).

In a multicenter randomized-controlled trial in major abdominal surgery patients, uncalibrated pulse contour analysis was used to define an optimal CI value after the induction of general anesthesia and before surgical incision (26). The post-induction preload optimized CI value was defined as the CI value that was observed when the PPV was less than 10% (either spontaneously or after fluid administration) and was used to trigger inotropic therapy with dobutamine during the intraoperative period (26). The use of this algorithm combining targets for PPV, CI, and mean arterial pressure resulted in a clinically relevant and statistically significant reduction in postoperative complications compared with the control group treated without PGDT (26).

A recently started study on individualized PGDT in major abdominal surgery patients (iPEGASUS^2^) uses a similar treatment algorithm, but a higher threshold for PPV (12%). The use of a higher PPV cutoff value [closer to the upper range of the “gray zone” (23)] represents a more restrictive approach to fluid administration.

## Conclusion

Perioperative goal-directed therapy protocols help to titrate fluids, vasopressors, or inotropes to predefined target values of hemodynamic variables in order to optimize global hemodynamics and eventually maintain or restore adequate oxygen delivery to the end-organs.

There is considerable evidence that PGDT can improve patient outcome in high-risk patients if both fluids and inotropes are administered to target hemodynamic variables reflecting blood flow.

Despite this evidence, PGDT strategies aiming at an optimization of blood flow seem to be not well implemented in routine clinical care.

The analysis of the arterial blood pressure waveform using invasive uncalibrated pulse contour analysis can be used to assess hemodynamic variables used in PGDT protocols. Pulse contour analysis allows the assessment of SV/CO and PPV/SVV and thus helps to titrate fluids and vasoactive agents based on principles of “functional hemodynamic monitoring.”

Pulse contour analysis-based PGDT treatment algorithms can be classified according to the hemodynamic variables they use as targets: PPV/SVV, SV/CO, or a combination of these variables. From a physiologic point of view, algorithms using both dynamic cardiac preload and blood flow variables as hemodynamic targets might be most effective in improving patient outcome.

Future research should focus on the improvement of hemodynamic treatment algorithms and on the identification of patient subgroups in which PGDT based on uncalibrated pulse contour analysis can improve patient outcome.

## Author Contributions

BS and DR conceived the study, performed the literature search, drafted the manuscript, and approved the final version of the manuscript to be published.

## Conflict of Interest Statement

BS collaborates with Pulsion Medical Systems SE (Feldkirchen, Germany) as a member of the medical advisory board and received honoraria for giving lectures and refunds of travel expenses from Pulsion Medical Systems SE. BS received institutional research grants, unrestricted research grants, and refunds of travel expenses from Tensys Medical Inc. (San Diego, CA, USA). BS received honoraria for giving lectures and refunds of travel expenses from CNSystems Medizintechnik AG (Graz, Austria). BS received research support from Edwards Lifesciences (Irvine, CA, USA). DR collaborates with Pulsion Medical Systems SE as a member of the medical advisory board and received honoraria for giving lectures and refunds of travel expenses from Pulsion Medical Systems SE. DR provided advising services for and received honoraria for giving lectures from Masimo (Masimo, Irvine, CA, USA) and Fresenius Kabi (Bad Homburg, Germany).

## Footnotes

^1^http://optimiseii.org.

^2^www.clinicaltrials.gov, identifier NCT03021525.
